# Highly Diverse, Poorly Studied and Uniquely Threatened by Climate Change: An Assessment of Marine Biodiversity on South Georgia's Continental Shelf

**DOI:** 10.1371/journal.pone.0019795

**Published:** 2011-05-25

**Authors:** Oliver T. Hogg, David K. A. Barnes, Huw J. Griffiths

**Affiliations:** British Antarctic Survey, Natural Environmental Research Council, Cambridge, United Kingdom; National Institute of Water & Atmospheric Research, New Zealand

## Abstract

We attempt to quantify how significant the polar archipelago of South Georgia is as a source of regional and global marine biodiversity. We evaluate numbers of rare, endemic and range-edge species and how the faunal structure of South Georgia may respond to some of the fastest warming waters on the planet.

Biodiversity data was collated from a comprehensive review of reports, papers and databases, collectively representing over 125 years of polar exploration. Classification of each specimen was recorded to species level and fully geo-referenced by depth, latitude and longitude. This information was integrated with physical data layers (e.g. temperature, salinity and flow) providing a visualisation of South Georgia's biogeography across spatial, temporal and taxonomic scales, placing it in the wider context of the Southern Hemisphere.

This study marks the first attempt to map the biogeography of an archipelago south of the Polar Front. Through it we identify the South Georgian shelf as the most speciose region of the Southern Ocean recorded to date. Marine biodiversity was recorded as rich across taxonomic levels with 17,732 records yielding 1,445 species from 436 families, 51 classes and 22 phyla. Most species recorded were rare, with 35% recorded only once and 86% recorded <10 times. Its marine fauna is marked by the cumulative dominance of endemic and range-edge species, potentially at their thermal tolerance limits. Consequently, our data suggests the ecological implications of environmental change to the South Georgian marine ecosystem could be severe. If sea temperatures continue to rise, we suggest that changes will include depth profile shifts of some fauna towards cooler Antarctic Winter Water (90–150 m), the loss of some range-edge species from regional waters, and the wholesale extinction at a global scale of some of South Georgia's endemic species.

## Introduction

**Figure 1:**
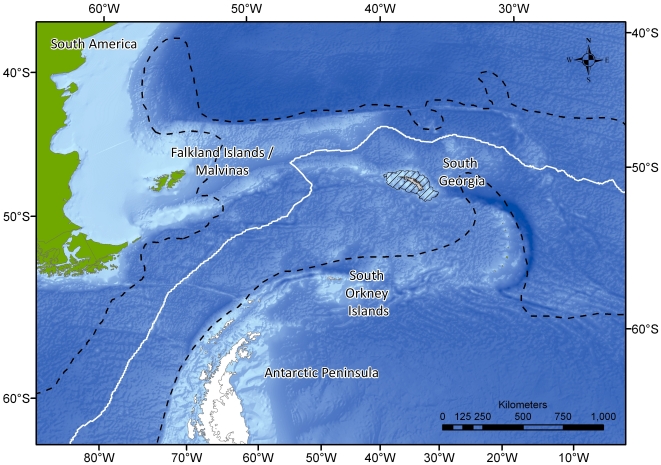
The position of South Georgia relative to the Polar Front (white line), and the Antarctic Circumpolar Current (black dashes).

The archipelago of South Georgia represents one of the largest, most isolated land masses and continental shelf areas in the Southern Ocean. Once situated adjacent to the Terra del Fuego region of South America [1], it is thought to have migrated to its current position 45–20 Ma [2], [3]. The region lies ∼1800 km to the east of the South American continental shelf (figure 1) bisecting the Antarctic Circumpolar Current (ACC). The Polar Front (PF) passes approximately 300 km to the north (mean distance derived from [4]) with the South ACC current, which transports nutrients and organisms (e.g. krill) from the Antarctic Peninsula, to the south [5]. The combination of this early separation from a continental land mass, a large shelf area, its high degree of geographic isolation and the proximity of nutrient rich currents represent important catalysts in the evolution of a biologically rich and distinct island, and identify South Georgia as a potentially important locality for biodiversity.

Studies of specific taxa [6]–[9] and multi-national collaboration in biodiversity databases such as SCARMarBIN [10] suggest South Georgia to be a key source of regional biodiversity, potentially supporting anomalously high levels of endemic and range-edge species (full definitions provided in materials and methods). In addition its waters support commercially important fisheries of Patagonian toothfish (*Dissostichus eleginoides*), mackerel icefish (*Champsocephalus gunnari*) and Antarctic krill (*Euphausia superb*). It may also be the most northern continental shelf with no known non indigenous marine species. Continental shelf biota is currently protected by a 22 km radial no-take zone and a 352 km^2^ management zone (figure 1) which restricts bottom fishing activities. Concurrent research is emerging however that identifies the near-surface waters around South Georgia as some of the fastest warming on earth [11]. Furthermore model projections suggest that over the coming decades the South Georgia will experience increased stress from ocean wide acidification [12].

With many species potentially at their thermal tolerance limit (reviewed in [13]), coupled with high levels of endemism, any drastic changes in environmental conditions may have severe impacts across scales to global biodiversity. Compounding this vulnerability is the fact that South Georgia's biota is generally Antarctic in character [9], [14]. As such it is characterised by slow growth, increased longevity and deferred sexual maturity [15] so consequently might find both toleration and adaptation difficult.

In 2002 a strategic plan was outlined as part of the Convention on Biological Diversity which, by 2010, aimed to achieve a “significant reduction” in the rate of biodiversity loss at regional, national and global levels (www.cbd.int/2010-target). This target was subsequently adopted by almost every nation as a political commitment central to the improvement of conservation, management and remedial practices [16]. Now in 2010 indications are that it is far from being met at a global level [17]–[19], with criticism levelled at the targets vagueness, as well as the timescale and baselines adopted [20]. One of the overriding problems identified is that in many key areas biodiversity was, and remains, to a large extent unquantified and consequently its loss cannot be measured let alone reduced. South Georgia is archetypal of this paucity in our knowledge of marine biodiversity and as such exemplifies the key failing of the 2010 CBD target whereby due to a lack of known baseline recordings the effects of environmental change are unquantifiable. In order to redress this situation an understanding of the structure and function of biodiversity, especially in ecologically sensitive areas such as South Georgia, is fundamental [21].

Considerable biodiversity data already exists for South Georgia but the majority of this data is scattered across literary sources (ISI journals and grey), in different institutes and languages. Much of such data may not have been checked taxonomically and most is not georeferenced in databases. In this paper we adopt a macroecological approach to collating, checking and mapping all available existing information onto the South Georgia shelf. As such it is the aim of this paper to create a thorough and accurate baseline measure of South Georgian marine biodiversity and thus provide a framework from which to identify ecologically sensitive areas and species, identify conservation priorities and monitor future biogeographical changes. This paper proposes to address four key questions: 1. How important is South Georgia as a source of regional and global biodiversity? 2. How important is it in terms of rare, endemic and range-edge species? 3. How is South Georgian biodiversity structured spatially and taxonomically? 4. Can we identify priority areas around South Georgia which are anomalously rich, vulnerable, or important to investigate due to paucity of knowledge?

## Results

### Biodiversity at South Georgia

**Figure 2:**
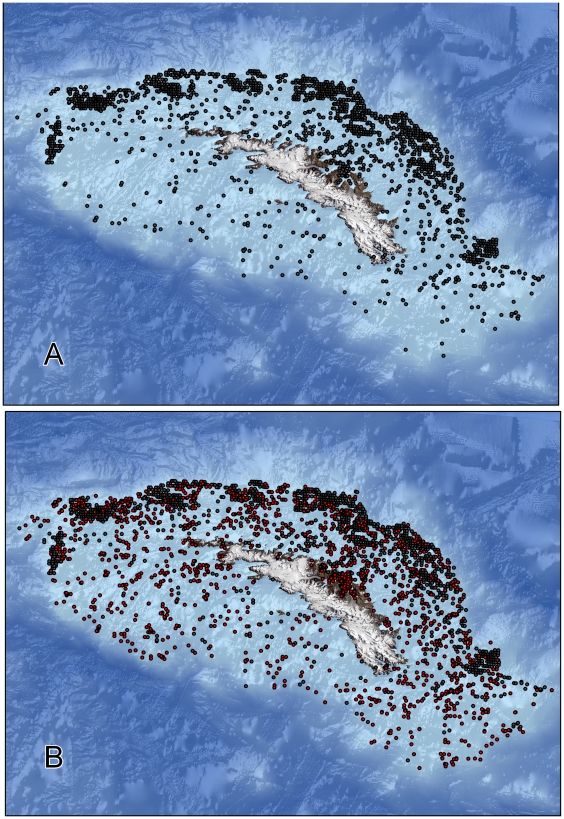
South Georgia Shelf sampling locations. (A) prior to the current study; (B) including data assimilated during this study (dots denoted in red).

Geo-referenced biodiversity data for South Georgia held in open access databases offered a relatively poor representation of known marine life around the island. Only six phyla were represented at the time of access, of which some such as Annelids had very few recorded species or specimens. Our collated data increased the number of records >5 fold, species 4 fold and sites, for which there is some information on biodiversity, by 90% (figure 2).

Marine biodiversity around South Georgia was rich across taxonomic levels; our data included representatives from 22 phyla, 51 classes and 436 families (see appendix S1 for full species list). The total number of individual specimens recorded was 17,732 comprising a total of 1,445 species. In terms of total number of individuals recorded the chordates and crustaceans were dominant with 8201 (46.2%) and 4767 (26.9%) of records respectively. In contrast no other phylum constituted more than 6.5% of overall records. In terms of richness, crustaceans were similarly well represented with 283 species (19.6%) but annelids, bryozoans, chordates, echinoderms, molluscs and nematodes all comprised 8–10% of total biodiversity (table 1).

**Table 1:** Species abundance and record counts across 22 recorded phyla.

Phylum	Species Count	Record Count
Crustaceans	283	4767
Nematodes	170	460
Annelids	147	725
Molluscs	161	588
Echinoderms	119	1160
Chordates	114	8201
Bryozoans	112	354
Chelicerates	93	530
Sponges	81	294
Cnidarians	78	358
Platyhelmenthes	33	52
Nemertea	15	60
Acanthocephala	12	19
Chaetognatha	5	92
Entoprocts	5	n/a
Sipuncula	4	55
Tardigrades	3	4
Brachiopods	3	3
Cephalorhyncha	2	4
Hemichordates	2	n/a
Ctenophora	2	n/a
Echiura	1	6
**Total**	**1445**	**17732**

**Figure 3:**
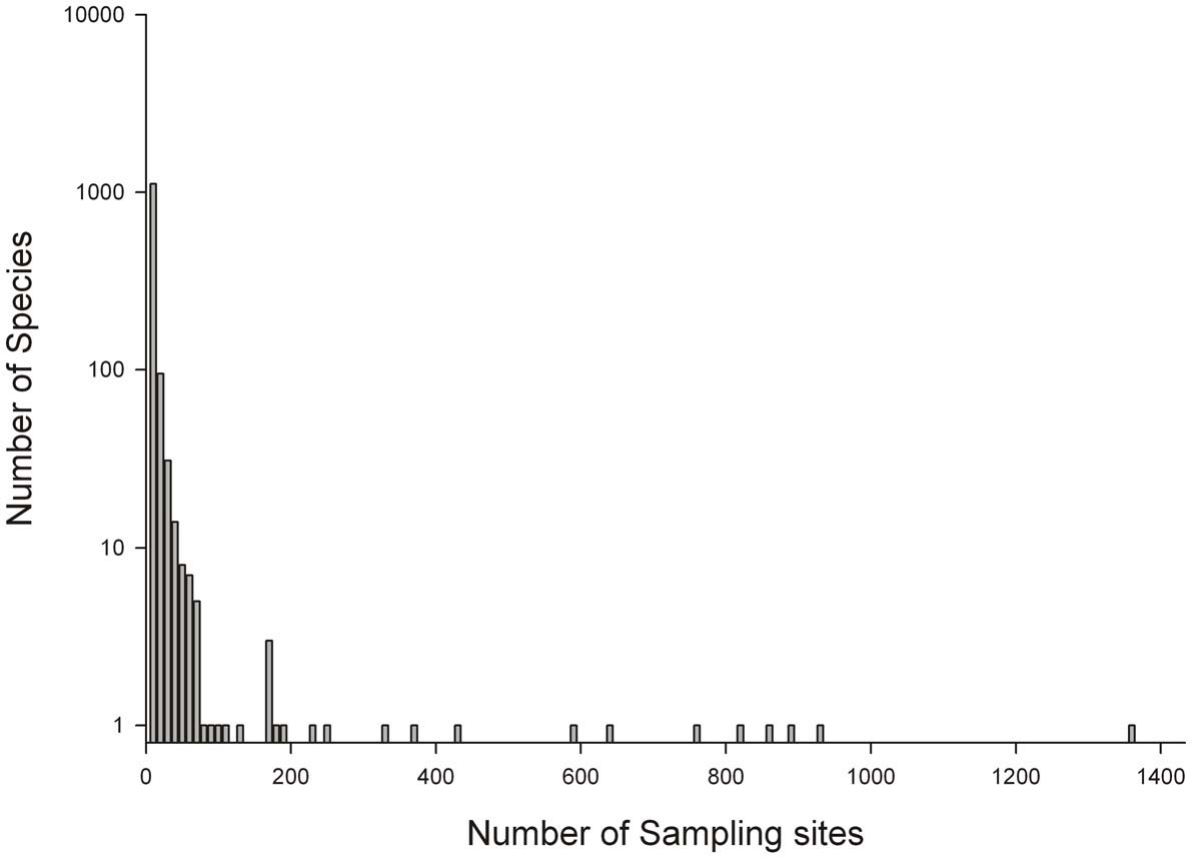
Species frequency on the South Georgian shelf. Species are ranked according to the number of distinct locations at which samples were records with the vast majority of species recording low record counts.

At species level 56% of records (9,961) were attributed to the 25 most abundant species, and 70% (12,472) to the 100 most abundant species. Commercially important species dominated the number of records, with a single species of Krill (*Euphausia superba*) forming 7.7% share of all records and 12 fish species collectively representing 40% of all records. Most species however were recorded from very few sites with 86% of species recorded in ≤10 samples across the whole study region (Figure 3) and 35% of species recorded only once.

### Endemism and Range Limits

Inclusion of the complete SCARMarBIN Southern Ocean dataset for biogeographical comparison suggested that many species are endemic or at range edges at South Georgia. We calculate that more than half of the bryozoan species and around 45% of cnidarians and molluscs are endemic to the South Georgia shelf. Levels of endemism did however vary considerably between phyla with the lowest proportions in the sponges and chordates (Table 2). Our data showed that South Georgia is a geographical range limit for a high proportion of the non-endemic shelf species, particularly northern range limits. For example 51.9% of cnidarians and 40% of molluscs have not been reported from lower latitudinal locations. With the notable exception of chordates, fewer species were found to be at southern range limits. The cumulative proportion of species recorded as endemic to, or on the edge of their geographical range at South Georgia was at, or approaching 100% for cnidarians and molluscs, 85% for bryozoans, 60% for crustaceans, with chordates and sponges at 30% and 24% respectively.

**Table 2:** Levels of endemism, and the proportion of species occurring at their northern and southern range limits at South Georgia.

Phylum	% Endemism	% Northern limit	% Southern limit
Bryozoans	55.6%	21.3%	8.3%
Cnidarians	44.2%	51.9%	3.9%
Molluscs^1^	45.9%	40.0%	13.3%
Crustaceans^2^	23.7%	29.0%	7.2%
Chordates^3^	8.5%	8.5%	12.8%
Sponges^4^	2.7%	17.6%	4.0%

Figures calculated from the ∼834, 800 records of known species distribution held within SCARMarBIN and recorded across six selected phyla. Due to either insufficient data collected at South Georgia or insufficient data held within SCARMarBIN some phyla from table 1 are omitted. Under sampled classes within included phyla are also omitted from analysis with such phyla denoted by the following suffixes to show the inclusion of:

1 Bivalves and Gastropods;

2 Malacostraca, Maxillopods and Pycnogonids;

3 Fish only;

4 Demosponges only.

### Biogeographical Trending

**Figure 4:**
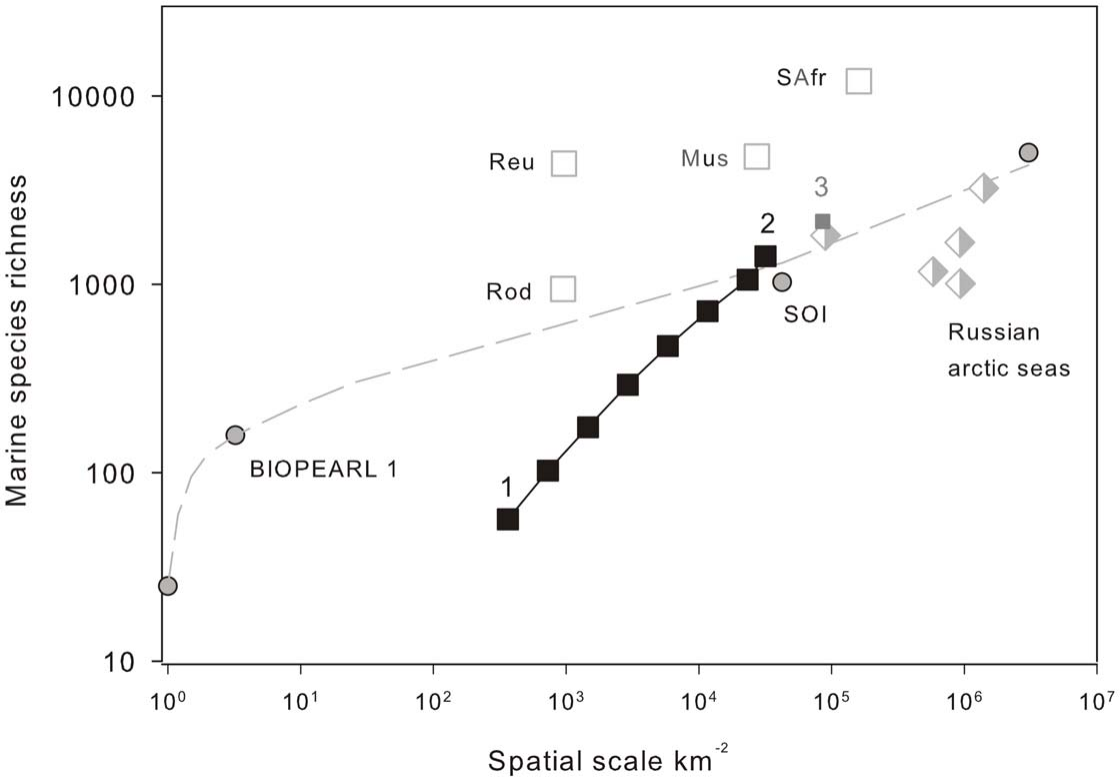
Marine Biodiversity over spatial scale. South Georgia data is denoted by filled black squares and presented in the format of 0.25×0.25° grid squares randomised through 999 iterations. Each successive point represents a doubling in shelf area from (1) a single 0.25×0.25° grid square, through to (2) the entire South Georgia Shelf. The combined biodiversity data from the South Georgian shelf and the South Orkney Islands (Barnes et. al. 2009), is represented by the filled grey square, point 3. Data for comparison was obtained from Barnes et. al. (2009) and includes biodiversity sampling from Reunion (Reu), Mauritius (Mus), Rodrigues (Rod), and South Africa (SAfr). Sampling from the Russian arctic seas (Sirenko *et al.*, 2001) is denoted by two tone diamonds. Grey circles denote data presented by Barnes et. al. (2009) for the South Orkney Islands, the first point representing biodiversity recorded from a randomly selected single trawl. Subsequent points record species richness for the entire BIOPEARL 1 expedition, total biodiversity for the South Orkney Islands (SOI) and finally reported species richness for the whole Southern Ocean (Clarke & Johnston, 2003).

**Figure 5:**
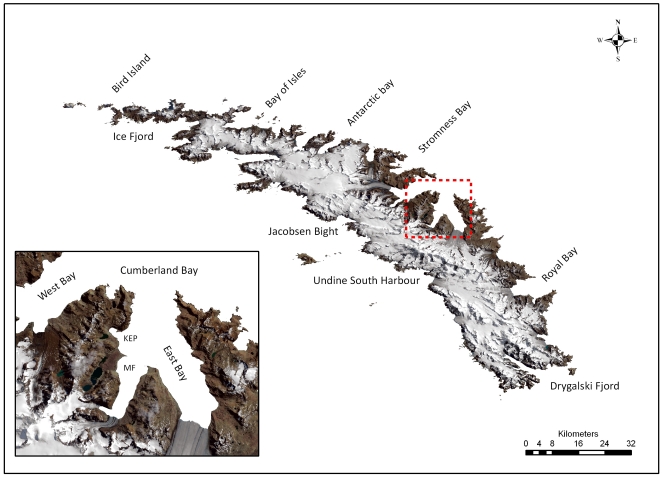
Map of South Georgia showing the main bays and islets around the island. The area of Cumberland Bay is enlarged in inset and includes the locations of King Edward Point (KP) and Moraine Fjord (MF).

**Figure 6:**
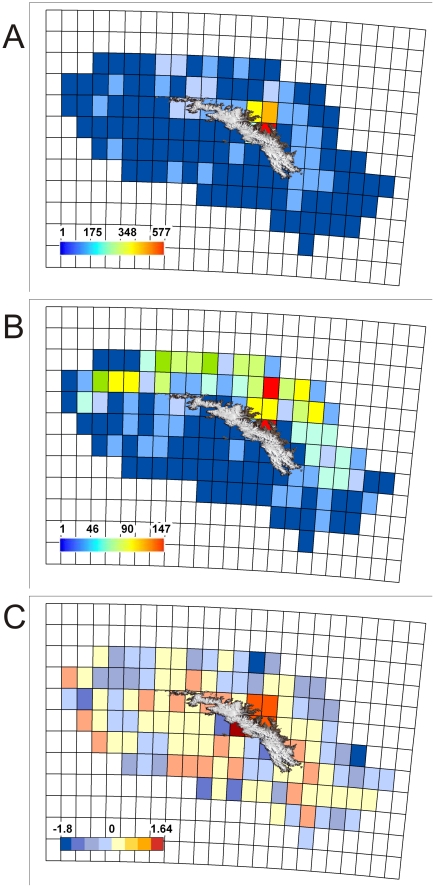
Total species and sampling distribution on the South Georgia shelf. (A) shows species richness; (B) Sampling intensity and (C) Linear regression residuals recorded in 0.25×0.25° grid squares across the South Georgia Shelf.

Analysis of marine biodiversity recorded over increasing spatial scale identified South Georgia as having a very spatially heterogeneous dataset, as evident from the consistently large increases in overall biodiversity observed through a systematic doubling of sampling scale (Figure 4). Subdivision of the South Georgia shelf into 0.25×0.25 degree grid squares supported these assertions, revealing a very geographically uneven distribution of species richness (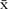 = 57.2; σ = 75.2). The most speciose grid occurred adjacent to the north coast at Cumberland Bay East (see figure 5) and contained 577 different species. Neighbouring grid squares to the north and west including areas of Cumberland Bay West and Stromness Bay also had species richness levels >400. Many other grids, especially along the south coast of the island and across the south and eastern shelf however, contained far fewer species with 75 grids totaling less than 50 species each (figure 6a).

**Figure 7:**
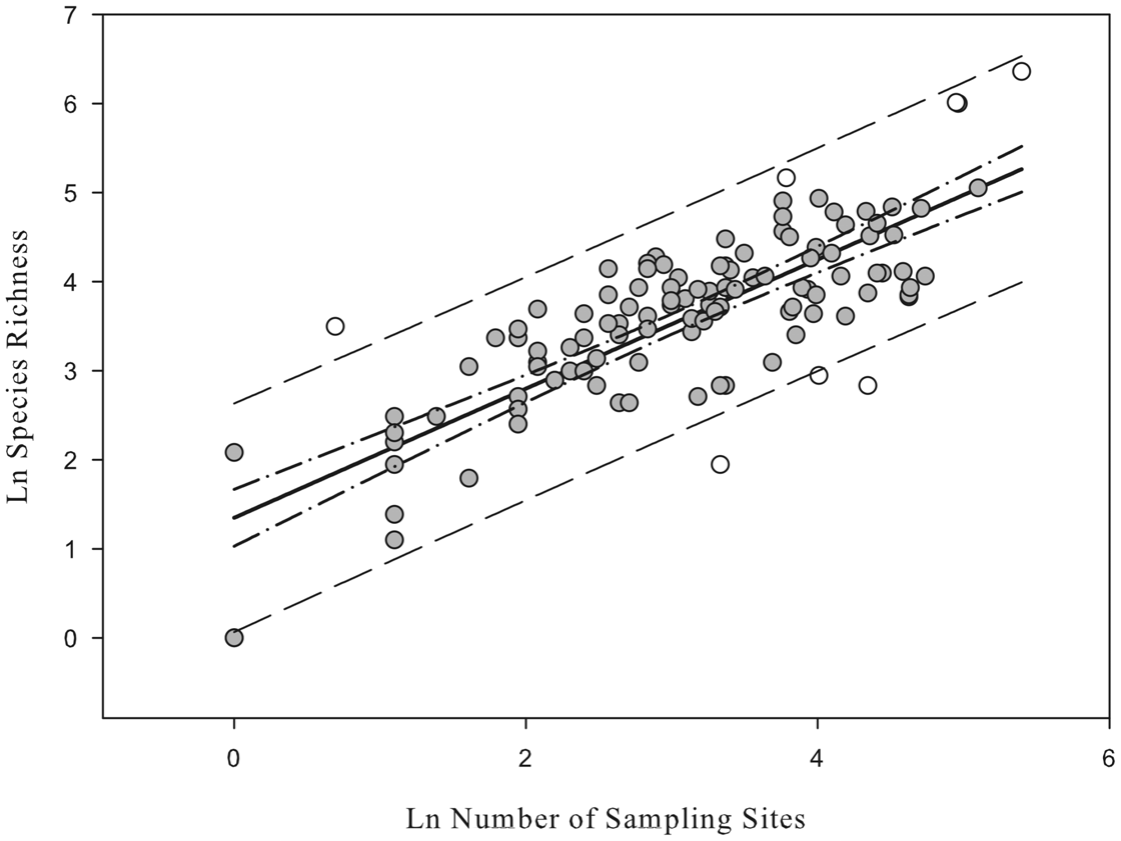
Relationship between sampling intensity and species richness. Each point represents a single 0.25×0.25° spatial grid on the South Georgia shelf. The regression line is shown in solid black, 95% regression confidence interval as dot/dash lines and 95% sample confidence lines as broad black dashing. Grid square with large residuals outside or approaching the 95% prediction line are represented by hollow circles.

In terms of sampling intensity, distribution was similarly geographically uneven (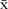 = 40.5; σ = 41.6). In some instances, notably at the above-mentioned areas around Cumberland Bay, high species numbers were coupled with high sampling intensity (figure 6b). Likewise, apparently species impoverished sites on the southern shelf were mainly characterized by low sample effort. Conversely however, many grids across the north and northwestern shelf breaks were intensive sampled (represented in figure 6b by yellow and green zones) but were not particularly species rich. Regression analysis on the logarithmic transformations of species and station counts allowed adjustment for the confounding influence of variance in sampling effort. Regression residual analysis (figure 7) revealed eight grid squares with anomalously high residuals. Two of the most anomalous residuals were recorded adjacent to each other in Jacobsen Bight East (1.64) and Jacobsen Bight West (−1.35) on the south coast of the island (figure 5c). The sampling effort of these sites however, was too low (1 and 2 samples respectively) to infer any meaningful biogeographic trend. Of the remaining grid squares Cumberland Bay and the waters immediately offshore from Cumberland Bay were identified as having large positive residuals whilst the biodiversity cold spots identified by negative residuals in figure 7 were located on the south and the northeastern shelf breaks. Comparatively high residuals were also recorded along the Western tip of South Georgia (near Bird Island), and at a number of mid southern shelf sites (represented as orange on figure 6c).

**Figure 8:**
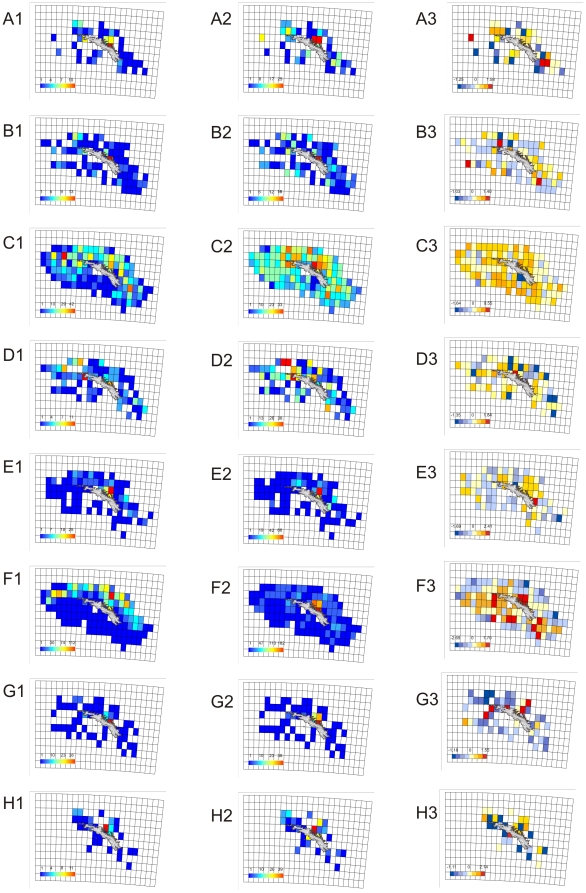
Species and sampling distribution of 8 phyla on the South Georgia shelf. (1) shows sampling intensity; (2) Species richness and (3) Linear regression residuals recorded in 0.25×0.25° grid squares across the South Georgia Shelf for (A) Bryozoans; (B) Cnidarians; (C) Chordates; (D) Echinoderms; (E) Molluscs; (F) Crustaceans; (G) Annelids and (H) sponges.

Sampling distribution varied considerably between phyla (Figure 8). Chordates and crustaceans were recorded in all but two grid squares on the shelf. Conversely, sampling of annelids, cnidarians and echinoderms was patchy, and bryozoan and sponge records were very patchy. This patchiness was particularly prevalent to the south and west of South Georgia, where we found no records of bryozoans in an area of shelf measuring 10,565 km^2^ (24.1% of total shelf area) nor sponges from an even larger area measuring 13,927 km^2^ (31.8% of total shelf area). Across the entire study area there were no bryozoans records from 66.1% of the shelf area or sponge records from 75.2% of the total area. This compared to 39.6% (for cnidarians), 34.7% (for molluscs) and just 2.5% (for crustaceans). Other large areas without records were for phyla with few species recorded species (such as brachiopods, echiurans, entoprocts, priapulans and tardigrades).

Collective trending of all phyla (figure 6) was not representative of patterns across many individual phyla (figure 8). Shelf edge locations tended to return negative residuals, especially along the North-East shelf break, and the area around Cumberland Bay tended to return positive residuals. Congruence between phyla across the rest of the shelf was however far more limited. Typically sampling was lower across the southern shelf but this did not translate in to similar patterns in residuals. Notably high south shelf residuals occurred in the crustaceans and cnidarians whereas the inverse was true for annelids and molluscs. Similarly the western tip of the Island was identified as relatively biologically rich in crustaceans, annelids and cnidarians but not obviously so for other phyla.

### Species Accumulation

**Figure 9:**
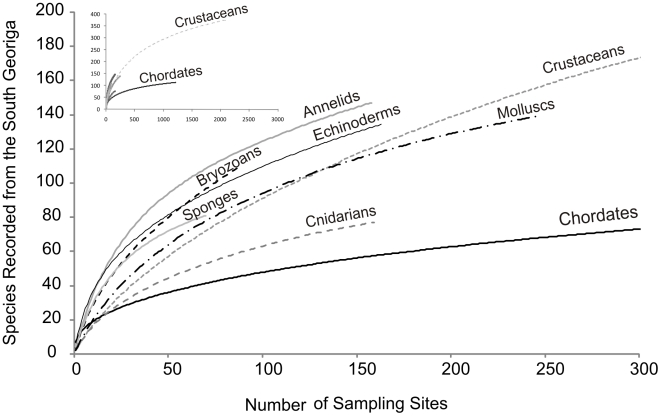
Rarefaction curves showing the rate of species accumulation with increasing sample effort in eight major taxa. Sample effort is defined by number of sampling sites. Inset presents the same data scaled to show trending of Crustaceans and Chordates over their entire sampling effort.

**Figure 10:**
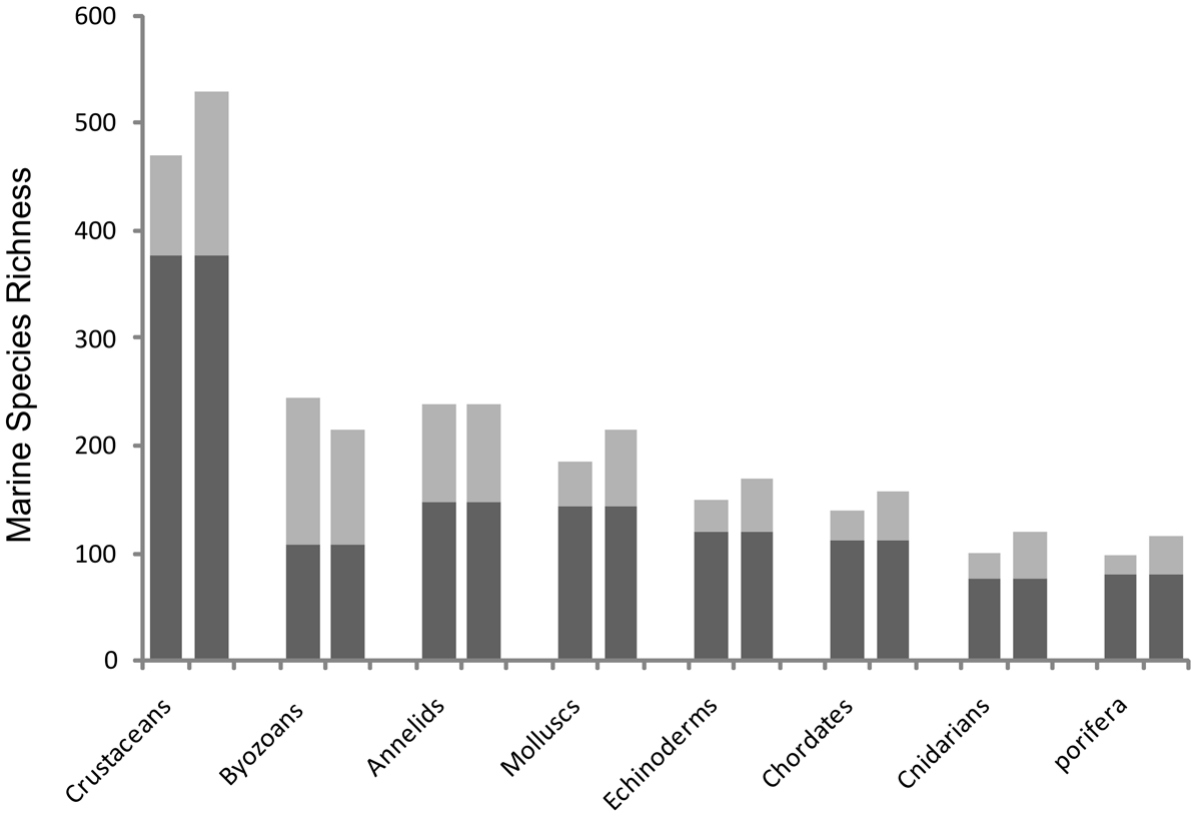
Species richness of eight major phyla on the South Georgia shelf. Dark grey bars show actual number of species recorded for each taxa; Light grey bars represent an estimated total species richness extrapolated using Chao 1(left hand column for each phyla) and Jacknife 2 (right hand column).

Rarefaction curves show the rate at which new species were accumulated with increasing sampling effort (Figure 9). In no phylum did such curves reach asymptote. The two most intensively recorded phyla, the chordates and crustaceans showed differentials of 0.02 and 0.05 new species per new sample site respectively. Echinoderms (0.38), sponges (0.35), and particularly bryozoans (0.67) retained high rates of species accumulation and as such represent the three phyla for which current biodiversity estimates are poorest. These trends were supported by Chao 1 and Jacknife 2 species richness estimators which respectively reported only 44% and 50% of probable bryozoans present at South Georgia as currently represented in our sampling. Comparatively, using the same estimators averaged across all eight major phyla 72.8% (σ = 13.3) and 65.8% (σ = 7.2) of estimated species richness was represented by our sampling). Extrapolations based on these eight phyla (representative of 82.6% of total species) place total species richness at 1,627 (Chao 1) and 1,760 (Jacknife 2). Relative variance in biodiversity between the major phyla with the exception of bryozoans remained relatively constant with rank order species richness remaining unchanged (Figure 10). Extrapolations based on the inclusion of all 22 recorded phyla at South Georgia however, produced much higher species estimates and a greater degree of variance between estimators with Chao 1 predicting 1,979 species compared to 2,366 estimated by Jacknife 2. Such large variances are unsurprising with the inclusion of under sampled minor phyla such as brachiopods (table 1) and phyla with geographically constricted sampling, as with nematodes.

## Discussion

### Southern Ocean Biodiversity

Previous attempts to frame Southern Ocean biodiversity within a global context indicate that in terms of species richness, arguably it is relatively impoverished [22]. This is exemplified through recent comparable studies undertaken in Japanese [23], Australian [24], New Zealand [25], Mediterranean [26], and Caribbean waters [27]. Conversely however, representation of global shelf species across many taxa is, at 8%, roughly in-line with the ∼8% of total global shelf area found in the Southern Ocean, [28]. Furthermore, unlike many lower latitudinal realms, our knowledge of many Southern Ocean taxa is relatively poor with high species accumulation rates across much of this polar region [29]. To quantify this, since 1993, the number of documented Southern Ocean shelf species has doubled to 8,800 [22], [30]. Even with this increase in our knowledge base it is nonetheless estimated that we have still only accounted for ∼50% of total Southern Ocean shelf biodiversity [31]. Our findings at South Georgia offer, by comparison a considerably better estimate of the biodiversity of a southern marine polar locality (67%±6%). Before drawing any meaningful conclusions from this study however we first assess the robustness of the data by evaluating how it is structured spatially and taxonomically.

### Spatial Distribution of Biodiversity

Sampling of the South Georgia shelf has been (by Southern Ocean standards) extensive, but over the past 125 years it hasn't benefited from a structured or uniform approach. This is not necessarily problematic when considering broad scale ecosystem level comparisons, such as between South Georgia and other polar archipelagos. It does however limit our understanding of how biodiversity is structured spatially across the South Georgia shelf itself. In some phyla for example, large areas of continental shelf remain completely un-sampled and for most phyla, in the areas that have been sampled, there is a heavy bias towards the waters in proximity to research bases such as at King Edward Point and the surrounding region of Cumberland Bay. Such biases can, in part, be accounted for by factoring in levels of sampling effort. In doing so, biodiversity (averaged across all taxa) was shown to be far from uniform across the shelf (figs. 4, 6c, and 7). Though there were no obvious biogeographic trends that showed ubiquity across all phyla, species richness tended to be higher coastally (especially on the northern side). One explanation for such a biogeographic skew could be that open shelf locations are probably comparatively more environmentally homogenous. In contrast coastal locations are likely to exhibit a higher degree of bathymetric heterogeneity, which in turn would drive variability in factors such as temperature, pressure, salinity, turbidity, wave/current exposure and light regimes. A cross section of environmental conditions is thus likely to create higher diversity in habitat types, a broader range of environmental niches, and as such a greater diversity of species.

We recognise however, that large amalgamations of diversity data, like reported here, are ultimately limited by the fact that they have been pooled from different sources. As such, though the data may be presented in the same format, the means by which the data has been obtained varies considerably. To illustrate consideration of sampling, we identify six well-sampled areas of the South Georgia shelf that purport to have anomalous levels of biodiversity. Of these, Cumberland Bay and the surrounding seas to the north are identified as very biologically rich, whilst shelf-break locations to the south and northeast were identified as very biologically poor. This may be a true reflection of biodiversity at these shelf locations. Conversely however the recording of biodiversity ‘cold’ spots may simply represent an artifact of sampling technique whereby areas that have experienced highly targeted sampling such as with exclusively pelagic trawling or selective benthic trawling inevitably record lower overall species counts. An improved understanding of South Georgia's biogeography must therefore ultimately come from a more even spread of sampling effort.

### South Georgia as a Source of Regional and Global Biodiversity

Direct comparisons between South Georgia and other Southern Ocean Islands are difficult because of the limited number of studies done at this intermediate level, i.e. between individual sampling stations and ocean scale interpolations. The South Orkney Islands (SOI) probably offers the best comparison with South Georgia as it is both relatively well sampled [32] and has a shelf area of the same order of magnitude. The SOI were recently reported to be richer in recorded marine biodiversity than many Atlantic, some Indian and even some Pacific archipelagos, such as Galapagos [32]. We report however that despite having a shelf area 75% that of the SOI, current available data identify the South Georgia shelf as supporting almost 40% more species. In addition, comparisons between species accumulation rates reveal known mega and macro-faunal richness at the SOI are unlikely to increase greatly with increased sampling [32]. In contrast general trends across taxa on South Georgia's shelf are of continually high rates of novel species identification. As such we argue that, with continued sampling effort, differences in marine biodiversity between the two archipelagoes will prove to be even more dissimilar than at present.

Elsewhere, Griffiths [22] showed Southern Ocean species counts to be highest along the North West Antarctic Peninsula, with a 3° by 3° grid around the South Shetland Islands supporting ∼700 species. This grid equates to a shelf area of approximately 51, 000 km^2^, (∼60% larger than the South Georgian Shelf). Based on this style of analysis, and with the inclusion of the data collated in this study we identify South Georgia as supporting over 3× more novel species per km^2^ than the Antarctic Peninsula.

In a global context, the data collected from South Georgia support inferences made by Barnes *et al.* [32], that Scotia arc localities can support equal or higher levels of biodiversity than equally well sampled sites at comparable northern latitudes [33], [34]. Indeed richness within bryozoans, sponges, nematodes and chelicerates was higher at South Georgia than in larger, and better sampled regions such as Hawaii [35] and the Baltic Sea [36].

South Georgia is geologically old and has a large shelf area. Positioned at the confluence of strong currents from the Antarctic and South American continental margins, it is supplied with both temperate and Antarctic species. Its geographic position has resulted in a relative lack of disturbance, being too north for significant ice scour and too far south to encounter high degrees of anthropogenic activity. These factors are probably all important drivers in high biodiversity at South Georgia. At present however the most anomalous feature of South Georgia's biodiversity is how well, by Southern Ocean standards, it has been studied making it very difficult to qualitatively compare.

### South Georgia as a Source of Rare and Endemic Species

The Southern Ocean is well documented as having some of the highest levels of marine endemism [37]. Though such estimates have been revised down recently [14], rates of 45–55% remain typical amongst many classes (e.g. pycnogonids, acideans, cyclostomes and cheilostome bryozoans) with levels increasing as high as ∼74% amongst gastropods [22]. To put these figures in a global context, average estimates of endemism in the Mediterranean are ∼20% [26], 25% in the Caribbean [27] and 33% around South Africa [38]. Contrary to ocean-level trends however, levels of regional endemism at localities within the Southern Ocean are, with the exception of the Weddell Sea and South Georgia, ubiquitously low [14].

The ability to report the level of endemism from South Georgia was constrained in some phyla by either inadequate sampling around the island, or more commonly inadequate reference sampling throughout the rest of the Southern Ocean. With the exclusion of these taxa however we found that South Georgian endemism amongst bryozoans, cnidarians and molluscs (gastropods and bivalves) was comparable to that of the Southern Ocean as a whole (46–56%) with fish and sponges tending to be more ubiquitous (3–8%). For both bryozoans and molluscs these findings represent a significant increase on previous estimates of shelf species endemism at South Georgia. Estimates of endemism amongst cheilostome bryozoans had previously stood at ∼15% [14], [21], a figure we now revise up to 56%. Likewise recorded endemism among bivalves and gastropods saw increases on previous estimates of 85% and 58% respectively. This high degree of local endemism to South Georgia goes further to polarising this archipeligo from trends in endemism found at other Antarctic localities.

Our findings support previous assertions [14] that South Georgia (along with the Weddell Sea) represents the exception to low rates of regional endemism south of the Polar Front.

Across a wider geographical context endemism amongst bryozoans was comparatively high; Griffiths *et al.* [14] reported significantly lower bryozoan endemism rates from all Antarctic and Sub-Antarctic islands as well as from New Zealand, Tasmania, South Africa and several regions of South America. These trends were not, however ubiquitous across all taxa at South Georgia. Molluscan endemism, though high, was considerably lower than recorded at other isolated islands such as Tristan da Cunha and the Kermadec Islands. Endemism in other groups such as amphipod crustaceans were comparable to other isolated islands, such as Galapagos, Tristan da Cunha and Society Islands but considerably lower than similarly sized New Caledonia [39]. Amongst fish, despite low species richness, endemism was high (∼9%) by most global comparisons, with exception of other extremely isolated shelves such as at Hawaii and Easter Islands [40].

The duration and the degree of isolation from other biogeographic zones are often cited as major drivers behind high levels of island endemism [37]. Both geologically old and geographically remote, South Georgia certainly seems to support this paradigm. Isolated from South America for ∼45 ma [2], [3] South Georgia is old enough for taxa to have evolved independently there, whilst remote enough (∼1800 km from the South American shelf) for the gene pool to be retained there. Isolation, be it geographical or environmental (as with the temperature gradient across the polar front) seems to be a pre-requisite for endemism. Insufficient isolation, as suggested by Barnes *et al.*, [32] for the SOI prevents any establishment of marine endemics. Within a marine environment however, what constitutes a sufficient level of isolation to support the retention of endemic species is a difficult point to quantify. At ∼600 km from the Antarctic Peninsula the SOI exhibited a relatively cosmopolitan biota [32]. Conversely however, Shag Rocks, only 240 km to the west of South Georgia is considered faunistically very distinct from its larger neighbour [41]. In a medium in which some propagules can travel across oceans [42] and non-pelagic larvae are known to disperse widely across the Scotia arc on kelp rafts [43] these boundaries of isolation go beyond a simple function of distance. Furthermore, the level of isolation at any given location will always be relative to the ranges of the taxonomic classes in question, and their ability to proliferate into neighboring regions. As such the endemic trends of some taxa are commonly shared across realms. Examples of this were reflected in our results in taxa that exhibit a predominantly benthic larval stage such as bryozoans [14], and at higher latitudes, gastropods [44].

In terms of species rarity, patterns of species abundance (figure 3) identify that few species recorded at South Georgia are common, and that most are rare. 496 species (34%) were shown to occur singularly and 1,244 species (86%) in 10 samples or less. This trend suggests that species abundance structure is qualitatively similar to both the Southern Ocean and elsewhere [29]. As such, though continued sampling is needed to understand finer level biogeographic trends, these broad macroecological patterns are likely to remain unchanged.

### South Georgia as a Range-Edge Species

A significant proportion of species recorded at South Georgia were shown to be at the edge of their known geographical ranges (table 2). For most of these species, South Georgia represented the northern most limit of their global distribution. Such trends had previously been identified at South Georgia amongst gastropods and bivalves [45]. Until now however, the degree to which range edge species dominate the species composition across a broad range of taxa at South Georgia had not been fully appreciated.

Species distributions have previously been shown to correlate closely with environmental temperature [46]–[48]. As such, temperature is widely cited as the most fundamental driver underlying the distribution limits of species [49]–[51]. The largest temperature cline in the Southern Ocean is the 3–4°C that differentiates either side of the Polar Front [52]. Indeed even for species with high tolerances to temperature variation, with the added consideration of the powerful eastward-flowing ACC, the PF represents a distinctive biogeographical discontinuity greatly limiting the exchange of epipelagic and benthic fauna [53]. Given South Georgia's position as the most northerly landmass (and shallow water shelf) south of the PF it is unsurprising that, for many Antarctic species that have proliferated as far as South Georgia, the island represents their most northerly extent. For some species however, South Georgia represents a southern range limit. Though these species were comparatively low in number they were broadly represented across all taxa (table 2). The distribution across the PF of highly directionally mobile taxa (e.g. fish) is perhaps unsurprising, and consistent with their physiology. In addition however, we report ubiquity across the PF in species of cheilostome bryozoans, crustaceans, gastropods, bivalves, demosponge, octocoral and pycnogonids.

Strong biogeographical links between Antarctica and South America are not a new concept [54], [55]. These links had, until now however, mainly been observed down to the level of family or genus [56] and largely considered an evolutionary legacy of the two regions shared provenance [57]. Clarke *et al.* [53] postulated that biogeographic patterns recorded in anomuran (spider) crabs [58], [59] demonstrated that a far greater degree of faunal exchange exists across the PF than had previously been accepted under the ‘isolated Southern Ocean paradigm’. Such exchange cannot be discounted as a mechanism for genetic flow to and from South Georgia especially given the known occurrence of kelp rafts [43], and driftwood [60], [61] as transport vectors. Considering the similarities between these different realms at family/genus level however, and the almost entirely benthic lifecycle of some of the aforementioned phyla (e.g. bryozoans). The most parsimonious explanation would seem to be that the presence of these species in both regions pre-existed the onset of the ACC ∼25 ma [62].

### Implications of Biogeographical Trends

Perhaps the most marked feature of South Georgian biodiversity is the cumulative dominance of species that are either endemic or at the limit of their geographical range. With 100% of cnidarians, 100% of molluscs, 80% of bryozoans and 60% of crustaceans recorded falling into this category we argue that the ecological implications of environmental change to the South Georgian marine ecosystem are potentially severe.

Sea surface temperatures around South Georgia have been identified as some of the fastest warming waters on earth, with the mean winter surface temperature (<100 m depth) increasing by ∼2.3°C in the 81 years preceding 2006 [11]. As a result of the hitherto lack of baseline biodiversity measurements at South Georgia the effects of these warming waters on South Georgia's fauna are non-quantifiable. Organisms are however limited in their responses. Some exhibit sufficient physiological plasticity to cope with changing environmental conditions. Those that do not must, adapt to the changing conditions, migrate to more optimal surroundings or go extinct [63].

Antarctic species have a more limited physiological tolerance to variations in temperature comparative to species elsewhere [64]. Adaptation is also more restricted in polar species due to slow development times, increased longevity and deferred sexual maturity [15]. With Antarctic benthic fauna developing at rates 5–10 times slower than their temperate counterparts [63], longer generation times result in a reduced probability of beneficial novel mutations. At South Georgia problems of response to regional warming are compounded because of the islands high number of endemic or range edge species. Endemic species, by definition are restricted in their ability to migrate to new locations. As such any inability to cope or adapt to warming waters would likely see the widespread loss of these species at both the regional level and also from the global system.

Antarctic species with South Georgia as their northern range edge for example would likely see their distribution contract towards the colder higher latitudes [45]. Conversely, species with South Georgia as their southern limit could proliferate beyond South Georgia, further into the warming Southern Ocean. At South Georgia itself this could see a wholesale change in the ecological dynamics of the ecosystem. Given the significantly larger proportion of northern range-edge species compared with southern range-edge the net result of these changes could be an overall reduction is South Georgian species richness.

There are several factors to consider at South Georgia that could limit the potentially adverse outcomes to this rapid sea warming. Firstly, as already mentioned South Georgia, with an annual surface sea temperature range of ∼5°C, has some of the largest variations in temperature south of the PF [65]. To put this figure in context, sea temperatures at the SOI have a maximum range of around 2.8°C [66], whilst in McMurdo Sound annual variations of <1°C are more typical [67]. One possible inference that could be made from these broad temperature ranges is that shallow shelf species at South Georgia have the ability to cope with large fluctuations in temperature not normally experienced elsewhere in the Southern Ocean. As such, assuming species at South Georgia aren't already pushing the envelope of their thermal tolerances it is possible that many already possess a predisposition to deal with large fluctuations in temperatures [68], [51].

Another consideration is the means by which migration to cooler waters can take place. Discussed so far is the ability of taxa to migrate to more favorable climes by lateral movement across geographical realms. Another option however is for more modest movements in depth. The Southern Ocean is characterised by three distinct summer water masses [69]: Surface Antarctic Water (SAW, 0–90 m), Winter Water (WW, 90–150 m), and Circumpolar Deep Water (CDW, >150 m). At South Georgia SAW typically reaches a summer maximum of 5°C [11], [70]; the CDW ranges from 1–3°C, whilst the intermediate layer of WW maintains temperatures as low as 0.5°C [71]. Across different latitudes the thermal regimes of these water masses change. On the Western Antarctic Peninsula for example temperatures of all three water masses are lower, with a divergence of over 4°C in SAW temperatures between the two localities [70]. In response to these temperature differentials, depth distributions in species have been shown to vary greatly between localities. Bivalve species occurring in the warmer SAW and CDW at the Antarctic Peninsula for example orientated themselves in the cooler WW at South Georgia [51]. If species exhibit depth profile shifts in response to thermal variability over latitudinal range, it seems probable the same could be true in response to thermal variability over a temporal range. As such we suggest that in response to rising surface water temperatures at South Georgia, species at their upper thermal limits in this zone could exhibit at shift in depth towards the cooler WW. Given the large proportion of northern range-edge species present at South Georgia the net result of such a widespread shift in depth ranges could be a higher degree of clustering of cold water Antarctic species at 90–150 m. The implications of this would be of great importance to any targeted conservation and management strategies at South Georgia.

### Conclusions

Over the past few decades attempts to understand the ramifications of anthropogenic activities on the structure of global biodiversity has moved to the forefront of scientific, political and social debate. Given the target driven nature of many of the interventions adopted to inform on these issues, it is perhaps surprising that most studies narrowly focus on specific taxa in order to infer more generalized patterns. By contrast this study adopted a macro-ecological approach to recording island level biodiversity, and as such represents the first attempt to map the biogeography of an archipelago south of the Polar Front.

We identify the South Georgian shelf as the most speciose region of the Southern Ocean recorded to date with greater species richness than comparable northern latitudes. Its marine fauna is marked by the cumulative dominance of endemic and range-edge species, many of which are potentially at their thermal tolerance limits. Consequently, our data suggests the ecological implications of environmental change to the South Georgian marine ecosystem could be severe. If sea temperatures continue to rise, one ramification could be the wholesale extinction at a global level of some of South Georgia's endemic species. Furthermore, we suggest that warming surface waters could also see depth profile shifts of some fauna towards cooler Antarctic Winter Water (90–150 m) and the complete loss of some range-edge species from South Georgian waters.

An improved understanding of South Georgia's biogeography, and its interactions with the surrounding oceans must ultimately come from a more even spread of sampling effort. It is intended however that the work presented in this study acts as a solid foundation for a more informed approach to conservation and management strategies at this isolated and vulnerable polar archipelago.

## Materials and Methods

### Study Area

The shelf area of South Georgia, which forms the focus of this paper, is here defined as waters adjacent to South Georgia less than 500 m in depth. Under this definition, and with the exclusion of the shelf surrounding Shag Rocks (an area considered faunistically distinct, e.g. [41]), the South Georgia Shelf comprises an area of approximately 31,800 km^2^.

Some environmental conditions around South Georgia are reasonably well described. Sea temperatures range from 0 to −1°C in winter and 3 to 4°C in summer on the upper shelf, which is amongst the warmest [70], and most variable [65] water mass inside the PF. Water temperature and other variables alter as much with depth as geography in the region [65]. The oceanography of the region is complex but sea temperature, salinity and current velocity and direction have been modeled at mesoscale by Young et al. [72] using a version of the Proudman Oceanographic Laboratory Coastal Ocean Modelling System. High resolution mapping of the seabed bathymetry around the island is in the process of being made available online by a South Georgia GIS project (http://www.sggis.gov.gs).

### Data Collection

Baseline biodiversity data was collected from a comprehensive review of reports and papers dating back to the German Polar Expedition of 1882, thus representing over 125 years of polar exploration. Significant information was obtained from some of the early research expeditions to South Georgia such as the Swedish Antarctic Expedition (1901–03), the Discovery cruises (1925–1938) along with more recent research trips including Islas Orcadas 575 (1975), USNS Eltanin (1968–1982), ANTARTIDA 8611 (1986–7), and BIOPEARL 1(2006). In addition records from the British Antarctic Groundfish Surveys (1986–2006) and Smithsonian Institute collection were also assimilated into the South Georgia dataset. Pre-existing open access databases, notably SOMBASE [73] and SCAR-MarBIN [10] acted as an important pool of South Georgia benthos data and also a significant catalyst to the initial development of the current study (complete biological data source list in appendix S2).

The scientific cruises, from which the data was drawn, differed in collection techniques and sampling effort. Most commonly Agassiz trawl and epi-benthic sledges were used but benthos was also collected using inshore SCUBA surveys, and by analysis of ROV video footage. This paper reports these findings in a standardized format recording all scientific classification to species level, depth at which specimens were recorded, and the location at which the specimens were found with the geo-reference linked to a Geographical Information System. Discrepancies in species classification were reconciled using the World Register of Marine Species (http://www.marinespecies.org) thus avoiding synonymies, which were especially prevalent in some of the older collections. In instances where data was collected using trawls of several km the start and end location of active trawling was recorded along with maximum and minimum depths. In instances where species were listed as present at South Georgia but were assigned no detailed biogeographical information, such data was included in general counts but not in biogeographical analysis (such species are listed in the appendix S1).

### Data Analysis

ArcGIS version 9.3 was used to visualize biodiversity data with sample points layered on top of geographical and bathymetric features. Analysis of sampling intensity and biological diversity was then quantified on a spatial scale by dividing the study area into a grid with each box representing 0.25 degrees of latitude by 0.25 degrees of longitude. Each box area varies with latitude (because our planet has near-spherical geometry), but with the South Georgia shelf spanning only 200 km at its widest point any change in area between boxes we considered to be negligible. Boxes were then scaled by sampling intensity (here defined as the number of unique latitude/longitude combinations sampled) and how speciose the box was (here defined as the count of distinct species). As a means of distinguishing true biodiversity hotspots from regions that had simply been extensively sampled, natural logarithmic transformations were carried out for species and station numbers from each grid square and regression analysis was carried out using Minitab, version 15 (Minitab Inc., State College, PA, USA). From this residuals were calculated for each grid square, which were then fed back into ArcGIS to produce a visual representation of the biodiversity ‘hot’ and ‘cold’ spots around South Georgia.

The rate of species accumulation and thus the degree to which our samples represent total marine biodiversity at South Georgia was analysed by rarefaction analysis, carried out in PRIMER (version 5). We used 999 iterations to produce species accumulation curves for each recorded major phylum. Differentials were then calculated to quantify the rate at which species discovery was still occurring. In order to estimate total species richness at South Georgia Estimate S, version 8.2 [74] was used to extrapolate the current trend of species accumulation using Chao 1 and Jacknife 2 as species richness estimators.

To analyse the geographical distribution of approximately 834,800 records from SCARMarBIN, database queries were run to assess which species had been sampled solely at South Georgia and could, within the confined of the dataset, be considered endemics. In addition species were also identified for which samples taken at South Georgia represented their southern or northern range limit, herein referred to as range-edge species.

## Supporting Information

Appendix S1 Census list of species present at South Georgia. Those with an * suffix were noted as present, but assigned no geographical location.
(XLS)

Appendix S2 Biological data source reference list.
(DOC)
