# Supplementary material for: Highly Diverse, Poorly Studied and Uniquely Threatened by Climate Change: An Assessment of Marine Biodiversity on South Georgia's Continental Shelf
Source: PLoS One. 2011 May 25;6(5):e19795. doi: 10.1371/journal.pone.0019795 (PMC3102052; doi:10.1371/journal.pone.0019795)
Supplement: Appendix S2 — Biological data source reference list. (DOC) [file pone.0019795.s002.doc]

APPENDIX S2: Biological data source reference list

Allgen CA (1959) Free-living marine nematodes, In: Further Zoological Results of the Swedish Antarctic Expedition 1901-1903, 5(2), 1-293.

Androsova EI (1972) Bryozoa Cheilostomata Anasca of the Antarctic and Subantarctic, In: Biological Reports of the Soviet Antarctic Expedition (1955-1958) 5, 315-345

Ärnbäck-Christie-Linde A (1938) Ascidiacea Part 1. In: Further Zoological Results of the Swedish Antarctic. Expedition. 3(4), 1-54.

Ärnbäck-Christie-Linde A (1950) Ascidiacea Part 2. In: Further Zoological Results of the Swedish Antarctic. Expedition. 4(4), 1-41.

Atkinson A (1989) Distribution of six major copepod species around South Georgia in early summer. Polar Biology 9, 353-363.

Atkinson A (1994) Diets and feeding selectivity among the epipelagic copepod community near South Georgia in summer. Polar Biology 14, 551-560.

Atkinson A, Peck JM (1988) A summer-winter comparison of zooplankton in the oceanic area around South Georgia. Polar Biology 8, 463-473.

Atkinson A, Ward P, Peck JM, Murray WA (1990) Mesoscale distribution of zooplankton around South Georgia. Deep-Sea Research 37, 1213-1227.

Atkinson A, Shreeve S, Pakhomov EA, Priddle J, Blight SP et al. (1996) Zooplankton responses to a phytoplankton bloom near South Georgia, Antarctica. Marine Ecological Progress Series 144, 195-210.

Atkinson A, Ward P, Hill A, Brierley AS, Cripps GC (1997) Krill-copepod interactions at South Georgia, Antarctica, I. Omnivory by Euphausia superb. Marine Ecology Progress Series, 160, 63-76.

Atkinson A, Ward P, Hill A, Brierley AS, Cripps GC (1999) Krill-copepod interactions at South Georgia, Antarctica, II. Euphausia superb as a major control on copepod abundance. Marine Ecology Progress Series, 176, 63-79

Augener H (1932) Antarktische und Antiboreale Polychaeten Nebst Einer Hirudinee. Scientific Results of the Norwegian Antarctic Expedition 1927-1928 1(9), 1-85.

Averincev VG (1972) Benthic Polychaetes Errantia from the Antarctic and Subantarctic collected by the Soviet Antarctic Expeditions, In: Biological Reports of the Soviet Antarctic Expedition (1955-1958) 5, 88-314

Balguerias E, Lopez Abellan LJ (1994) On the Presence of Paralomis spinosissima and Paralomis Formosa in catches taken during the Spanish survey Antartida 8611. CCAMLR Science 1, 165-173.

Balguerias E, Bruno J, Cestino JM, Daroca E, Gil de Sola L et al. (1989) Biologia Pesquera. In: Informe de Resultados Antartida 8611, pp. 267-483. Madrid.

Banks N (1914) Arachnida from South Georgia, In: A report on the South Georgia expedition. Museum of the Brooklyn Institute of Arts and Sciences, Science Bulletin 2(4), 78-80.

Barnard KH (1930) Crustacea II. Amphipoda. Natural History Report of the British Antarctic Terra Nova Expedition 1910, 8, 307-454.

Barnard KH (1932) Amphipoda. Discovery Report 5, 1-326.

Barnes DKA, Griffiths HJ, Kaiser S (2009) Geographic range shift responses to climate change by Antarctic benthos: where we should look. Marine Ecology Progress Series, 393, 13-26.

Bergenhayn JRM (1937) Antarktische und Subantarktische Polyplacophoren. Sci. Res. Norwegian Antarctic Expedition 1927-1928 1(17), 1-12.

Block W, Somme L (1984) Ecophysiology of two intertidal mites at South Georgia. Oikos 42(3), 276-282.

Broch H (1948) Antarctic Hydroids. Scientific Results of the Norwegian Antarctic Expedition 1927-1928 1(28), 1-23.

Burchett MS (1983) Abundance of the nearshore fish population at South Georgia (Antarctica) sampled by Trammel Net. British Antarctic Survey Bullitin 61, 39-43.

Cairns SD (1983) Antarctic and Subantarctic Stylasterina (Coelenterata: Hydrozoa). Biology of the Antarctic Seas 8, In: Antarctic Research Series 38, 61-164.

Capitanio FL, Daponte MC, Esnal GB (2003) The Classification of Antarctic Appendicularians: the Oikopleura gaussica group. Antarctic Science 15(4), 476-482.

Carlgren O (1930) Antarctic and Subantarctic Actinaria. Scientific Results of the Norwegian Antarctic Expedition. 1927-1928 and 1928-1929, 1(7), 1-6.

Casas C, Ramil F, Van Ofwegen LP (1997) Octocorallia (Cnidaria:Anthozoa) from the Scotia Arc, South Atlantic Ocean. I. The Genus Alcyonium Linnaeus, 1758. Zool. Med. Leiden 71, 299-311.

Clarke A (1977) Seasonal variation in the total lipid content of Chorismus Antarcticus (Pfeffer) (Crustacea: Decapoda) at South Georgia. Journal of Experimental Biology and Ecology 27, 93-106.

Clarke A. Lakhani KH (1979) Measures of biomass, moulting behaviour and the pattern of early growth in Chorismus antarcticus. British Antarctic Survey Bullitin 47, 61-88.

Collins MA, Allcock AL, Belchier M (2004) Cephalopods of the South Georgia slope. Journal of Marine Biological Association UK 84, 415-419.

Collins MA, Shreeve RS, Fielding S, Thurston MH (2008) Distribution, growth, diet and foraging behaviour of the yellow-Fin Notothen Patagonotothen guntheri (Norman) on the Shag Rocks Shelf (Southern Ocean). Journal Fish Biology 72, 271-286.

Collins MA, Yau C, Guilfoyle F, Bagley P, Everson I et al. (2002) Assessment of stone crab (Lithodidae) density on the South Georgia slope using baited video cameras. Journal of Marine Science 59, 370-379.

Cruz M, Gabor N, Mora L, Jimenez R, Mair J (2003) The known and unknown about marine biodiversity in Ecuador (continental and insular). Gayana 67, 232-260.

Daly HI, Rodhouse PG (1994) Comparative morphology of two sympatric Pareledone species from South Georgia. Antarctic Science 6(2), 163-169.

Davenport J (1995) Upwelling-generated plankton strandlines: important predictable food sources for seabirds at Husvik, South Georgia. Marine Biology 123, 207-217.

Davenport J, Macalister H (1996) Environmental conditions and physiological tolerances of intertidal fauna in relation to shore zonation at Husvik, South Georgia. Journal of the Marine Biological Association UK 76, 985-1002.

De Broyer C, Lowry JK, Jazdzewski K, Robert H (2009) Census of Antarctic marine life, synopsis of the amphipoda of the Southern Ocean Vol 1: Catalogue of the gammaridean and corophiidean amphipoda (crustacea) of the Southern Ocean with distribution and ecological data. Bulletin de l’institut Royal des Sciences Naturelles de Belgique Biologie, 77.

Dell RK (1955) The occurrence of Priapulus in New Zealand waters. Transcriptions of the Royal Society. Of New Zealand 82(5), 1129-1133.

Dell RK (1972) Antarctic Benthos. Advances in Marine Biology 10, 1-216.

Dodge JD, Priddle J (1987) Species composition and ecology of dinoflagellates from the Southern Ocean near South Georgia. Journal of Plankton Research 9(4), 685-697.

Ekman S (1925) Holothurien. In: Further Zoological Results of the Swedish Antarctic Expedition 1901-1903 1(6), 1-194.

Emschermann P (1993) On Antarctic Entoprocta: nematocyst-like organs in a loxosomatid, adaptive development strategies, host specificity, and bipolar occurrence of species. The Biological Bulitin. 184, 153-185.

Foster MW (1974) Recent Antarctic and Subantarctic brachiopods. Antarctic Research Series 21, 1-189.

Garcia FJ, Troncoso JS, Garcia-Gomez JC, Cervera JL (1993) Anatomical and taxonomical studies of the Antarctic nudibranchs Austrodoris kerguelenesis (Bergh, 1884) and A. Georiensis n. Sp. from Scotia Sea. Polar Biology 13, 417-421.

Gorny M (1999) On the biogeography and ecology of the Southern Ocean decapod fauna. Scientia Marina, 63, 367-382.

Grieg JA (1929) Echinodermata. Scientific Results of the Norwegian Antarctic Expedition 1927-1928 and 1928-1929, 1(2), 1-16.

Griffiths H J, Linse K, Barnes DKA (2008) Distribution of macrobenthic taxa across the Scotia Arc, Southern Ocean. Antarctic Science, 20(3), 213-226.

Hartman O (1953) Non-pelagic Polychaeta. Further Zoological Results of the Swedish Antarctic Expedition 1901-1903 4(2), 1-11.

Hardy AC, Gunther ER (1935) The plankton of the South Georgia whaling grounds and adjacent waters 1926-1927. Discovery Reports 11, 1-456.

Hartman O (1953) Non-pelagic polychaeta. Further Zoological Results of the Swedish Antarctic Expedition 1901-1903, 4(2).

Hartman O (1967) Polychaetous annelids collected by the USNS Eltanin and Staten Island cruises, chiefly from Antarctic Seas. Allan Hancock Monographs, In: Marine Biology, 2, 1-387.

Hastings AB (1943) Polyzoa (Bryozoa), Discovery Reports, 22, 301-510.

Hayward PJ (1990) Some Antarctic and sub-Antarctic species of Smittinidae (Bryozoa: Cheilostomata). Journal of the Zoological Society London 222, 137-175.

Hayward PJ (1993) New species of cheilostomate bryozoa from Antarctica and the Sub Antarctic Southwest Atlantic. Journal of Natural Hisory 27.

Hayward P J, Ryland J S (1990) Some Antarctic and Sub Antarctic Species of Microporellidae (Bryozoa: Cheilostomata). Journal of Natural History 24, 1263-1287.

Hayward P J, Thorpe J P (1989) Systematic note on some Antarctic Ascophora (Bryozoa, Cheilstomata). Zoologica Scripta, 18(3), 365-374.

Hofmann EE, Klinck JM, Locarnini RA, Fach BA, Murphy EJ (1998) Krill transport in the Scotia sea and environs. Antarctic Science 10, 406-415.

Ingels S, Vanhove S, De Mesel I, Vanreusel A (2006) The biodiversity and biogeography of the free-living nematode genera Desmodora and Desmodorella (family Desmodoridae) at both sides of the Scotia Arc. Polar Biology 29, 936-949.

Jäderholm E (1905) Hydroiden aus Antarktischen und Subantarktischen meeren. Wissenschaftliche Ergebnisse der Schwedischen Südpolar-Expedition 1901-1903, 5(8), 1-42.

John DD (1938) Crinoidea. Discovery Reports 18, 121-222.

Karling TG (1952) Kalyptorhynchia (Turbellaria). Further Zoological Results Swedish Antarctic. Expedition 1901-1903 4(9), 1-50.

Karling TG (1973) Anatomy and taxonomy of a new Ocoplanid (Turbellaria, Proseriata) from South Georgia. Mikrofauna des Meeresboden, 16, 361-369.

Karling TG (1977) Taxonomy, phylogeny and biogeography of the genus Austrorhynchus Karling (Turbellaria, Polycystididae). Mikrofauna des Meeresboden, 61, 153-165.

Kawaguchi S, Siegel V, Litvinov F, Loeb V, Watkins J (2004) Salp distribution and size composition in the Atlantic sector of the Southern Ocean. Deep-Sea Research part II, 51, 1369-1381.

Kornicker LS (1983) Biology of the Antarctic Seas 8. Antarctic Research Series, 38, 1-436.

Kott P (1969) Antarctic Ascidiacea. Antarctic Research Series 13, 1-239

Kott P (1971) Antarctic Ascidiacea II. Antarctic Research Series 17, 11-82.

Kramp PL (1949) Medusae and siphonophora. Scientific Results of the Norwegian Antarctic Expedition. 1927-1928, 1(30), 1-8.

Kudenov JD (1992) Amphinomidae and Euphrosinidae (Annelida: Polychaeta) principally from Antarctica, the Southern Ocean, and Subantarctic regions. Biology of the Antarctic Seas XXII. Antarctic Research Series 58, 93-150.

InglesJ, Vanhove S, De Mesel I, Vanreusel (2006) The biodiversity and biogeography of the free-living nematode genera Demodora and Demodorella (family Desmodoridae) at both sides of the Scotia Arc. Polar Biology 29, 936-949.

Loman JCC (1923) The Pycnogonida of the Swedish Antarctic Expedition (1901-1903). In: Further Zoological Results of the Swedish Antarctic Expedition 1901-1903 1(2), 1-39.

Lopez Abellan LJ, Balguerias E (1994) On the presence of Paralomis spinosissima and Paralomis formosa in catches taken during the Spanish survey Antartida 8611. CCAMLR Science, 1, 165-173.

Lopez de la Cuadra CM, Garcia Gomez JC (2000) The cheilostomate bryozoa (Bryozoa: Cheilostomatida) collected by the Spanish ‘Antartida 8611’ Expedition to the Scotia Arc and South Shetland Islands. Journal of Natural History 34, 755-772.

Meyer MC, Burreson EM (1990) Some Leeches (Hirudinea: Piscicolidae) of the Southern Oceans. Biology of the Antarctic Seas 11, In: Antarctic Research Series, 52, 219-236.

Millar RH (1960) Ascidiacea. In: Discovery Reports 30, 1-160.

Monniot C, Monniot F (1983) Ascidies Antarctiques et Subantarctiques: Morphologie et Biogeographie. Memoirs du Museum National d'Histoire Naturelle, Nouvelle, Series A Zoologie, 125, 1-168.

Monro CAA (1930) Polychaete Worms. Discovery Reports. 2, 1-222.

Mortensen TH (1918) The Crinoidea. Der Schwedischen Sudpolar-Expedition 1901-1903 6(8) 1-23.

Newman WA, Ross A (1971) Antarctic Cirripedia. Antarctic Research Series 14, 1-257.

Nilsson-Cantell CA (1930) Thoracic Cirripedes collected in 1925-1927. Discovery Reports 2, 223-260.

Nilsson-Cantell CA (1939) Thoracic Cirripedes Collected in 1925-1936. Discovery Reports, 18, 223-238.

Pagès F, Orejas C (1999) Medusae, Siphonophores and Ctenophores of the Magellan Region. Scientia Marina 63, 51-57.

Pakhomov EA, Verheye HM, Atkinson A, Laubscher RK, Taunton-Clark J (1997) Structure and grazing impact of the mesozooplankton community during late summer 1994 near South Georgia, Antarctica. Polar Biology 18, 180-192.

Peña Cantero AL, García Carrascosa AM (1999) Biogeographical distribution of the benthic Thecate hydroids collected during the Spanish “Antartida 8611” Expedition and comparison between Antarctic and Magellan benthic hydroid faunas. Scientia Marina, 63, 209-218.

Peña Cantero AL, García Carrascosa AM (1999) Two new species of Staurotheca Allman, 1988 (Cnidaria, Hydrozoa, Sertulariidae) from the Scotia Sea (Antarctica). Polar Biology 21, 155-165.

Peña Cantero AL, Gili JM (2006) Benthic hydroids (Cnidaria, Hydrozoa) from off Bouvet Island (Antarctic Ocean). Polar Biology 29, 764-771.

Pfeffer G (1887) Die Krebse von Sud-Georgien Nach der Ausbeute der Deutschen Station 1882-83. Jahrbuch der Hamburg, pp. 43-150.

Platt HM (1979) Ecology of King Edward Cove, South Georgia: Macro-Benthos and the Benthic Environment. British Antarctic Survey Bulletin 49, 231-238.

Pond D, Watkins J, Priddle J, Sargent J (1995) Variation in the lipid content and composition of Antarctic krill Euphausia superb at South Georgia. Marine Ecology Progress Series, 117, 49-57.

Priddle J, Heywood RB, Theriot E (1986) Some environmental factors influencing phytoplankton in the Southern Ocean around South Georgia. Polar Biology 5, 65-79.

Pugh PJA (1993) A synonymic catalogue of the Acari from Antarctica, the sub-Antarctic Islands and the Southern Ocean. Journal of Natural History 27, 323-421.

Pugh PJA (1993) A synonymic catalogue of the Acari from Antarctica, the Sub-Antarctic Islands and the Southern Ocean. Journal of Natural History, 27, 323-421.

Pugh PJA (1995) Air-breathing littoral mites of Sub-Antarctic South Georgia. Journal of the Zoological Society London, 236, 649-666.

Pugh PJA, Bartsch I (1993) Ecology of the Littoral Halacaridae (Acari: Prostigmata) of South Georgia in the Sub-Antarctic. Journal of Natural History, 28, 75-85.

Pugh PJA, Convey P (2000) Scotia Arc Acari: Antiquity and Origin. Zoological Journal of the Linnean Society of London, 130, 309-328.

Pugh PJA, Dartnall HJG (1994) The Acari of fresh and brackish water habitats in the Antarctic and Sub-Antarctic Regions. Polar Biology, 14, 401-404.

Pugh PJA, MacAlister HE (1994) Acari of the supralittoral zone on Sub-Antarctic South Georgia. Pedobiologia 38, 552-565.

Richters F (1920) Moosbewohner, In: Schwedische Sudpolar-Expedition 1901-1903, 6, 1-16.

Rogick MD (1965) Biogeography and ecology in Antarctica: Bryozoa of the Antarctic. Monographiae Biologicae, 15, 401-413.

Rota E, Erseus C (1997) A re-examination of Grania monochaeta (Michaelsen) (Oligochaeta: Enchytraeidae), with descriptions of two new species from Antarctic South Georgia. Journal of Natural History, 31, 27-42.

Rustad D (1930) Mysidacea. Scientific Results of the Norwegian Antarctic Expedition 1927-1928 and 1928-1929. 1(6), 1-28.

Shoemaker CR (1914) Amphipods of the South Georgia expedition, In: A Report on the South Georgia Expedition. Museum of the Brooklyn Institute of Arts and Sciences, Science Bulletin, 2(4), 73-77.

Shreeve RS, Collins MA, Tarling GA, Main CE, Ward P, et al. & (2009). Feeding ecology of myctophid fishes in the northern Scotia Sea. Marine Ecological Progress Series, 386, 221-236.

Skigsberg T (1920) Studies of Marine Ostracods, In: Zoologiska Bidrag. Zoologische Beitrage aus Uppsala.

Sømme L, Block W (1984) Ecophysiology of two intertidal mites at South Georgia. Oikos 42(3), 276-282

Sømme L, Block W (1986) Ecophysiology of intertidal mites at South Georgia. Colloque sur les Ecosystemes Terrestres Subantarctiques 58, 107-109.

Soot-Ryen T. (1951) Antarctic Pelecypods. . Results of the Norwegian Antarctic Expedition 1927-1928, 1(32), 1-46.

Stephen AC (1941) The Echiuridae, sipunculidae and priapulidae collected by the ships of the Discovery committee during the years 1926 to 1937. Discovery Reports, 21, 235–260.

Stephenson J (1932) Oligochaeta, Part 1: microdrili. Discovery Reports, 4, 233-264.

Støp-Bowiz, C (1949) Polychetes Pelagiques. Scientific Results of the Norwegian Antarctic Expedition 1927-1928, 1928-1929, and 1930-1931, 31, 1-25.

Tattersall OS (1955) Mysidacea. Discovery Reports 28, 1-190.

Tebble N (1960) The distribution of pelagic polychaetes in the Southern Atlantic Ocean. Discovery Reports, 30, 161- 300.

Thstje S, Hall S, Hauton C, Held C, Tyler P (2008) Encounter of lithodid crab Paralomis birsteini on the continental slope off Antarctica, sampled by ROV. Polar Biology, 31, 1143-1148.

Vigeland I (1952) Antarctic Bryozoa. Scientific Results of the Norwegian Antarctic Expedition 1927-1928, 1(34), 1-15.

Vladimirskaya YV (1978) Distribution of zooplankton in the vicinity of South Georgia Island in the summer of 1975. Oceanology 18(3), 340-342.

Ward P (1989) The distribution of zooplankton in an Antarctic fjord at South Georgia during summer and winter. Antarctic Science 1(2), 141-150.

Ward P (1985) On the Biology of Antarctomysis Ohlini (Crustacea: Mysidacea) at South Georgia. British Antarctic Survey Bulletin. 67, 13-23.

Ward P, Atkinson A, Murray AWA, Wood AG, Williams R, Poulet SA (1995) The summer zooplankton community at South Georgia: biomass, vertical migration and grazing. Polar Biology, 15, 195-208.

Ward P, Shreeve RS (1999) The Spring Mesozooplankton Community at South Georgia: a Comparison of Shelf and Oceanic Sites. Polar Biology, 22, 289-301.

Ward P, Shreeve R, Tarling GA (2006) The autumn mesozooplankton community at South Georgia: Biomass, population structure and vertical distribution. Polar Biology, 29, 950-962.

Ward P, Wood AG (1988) The distibution of the Euchaetidae (Copepoda:Calanoida) around South Georgia. Polar Biology 9, 45-52.

Watts J, Thatje S, Clarke S, Belchier M (2006) A description of larval and early juvenile development in Paralomis spinosissima (Decapoda: Anomura: Paguroidea: Lithodidae) from South Georgia waters (Southern Ocean). Polar Biology, 29, 1028-1038.

Westblad E (1952) Turbellaria (Excl. Kalyptorhynchia). Further Zoological Results of the Swedish Antarctic Expedition 1901-1903 4(8), 1-55.

Wheeler JFG (1934) Nemerteans from the south Atlantic and Southern Oceans. Discovery Reports, 9, 217–294.

Yau C, Allcock AL, Daly HI, Collins, MA (2002) Distribution of Pareledone Spp. (Octopodidae Eledoninae) around South Georgia. Bullitin of Marine Science, 71(2), 993-1002.

Yau C, Collins MA, Bagley PM, Everson E, Priede IG (2002) Scavenging by megabenthos and demersal fish on the South Georgia slope. Antarctic Science 14, 16-24.

Zdzitowiecki K (1987) Acanthocephalans of marine fishes in the regions of South Georgia and South Orkneys (Antarctic). Warszawa 15(6), 211-217.

Zdzitowiecki K (1990) Occurrence of Acanthocephalans in fishes of the open sea off the South Shetlands and South Georgia (Antarctic). Acta Parasitologica Polonica 35(2), 131-141.

Zdzitowiecki K, White GW (1992) Digenean Trematoda infection of inshore fish at South Georgia. Antarctic Science, 4(1), 51-55.

Zdzitowiecki K, White GW (1992) Acanthocephalan infection of inshore fish in two fjords at South Georgia. Antarctic Science 4(2), 197-203.

Zdzitowiecki K, White GW (1996) Acanthocephalan infection of inshore fish at the South Orkney Islands. Antarctic Science 8(3), 273-276.

Zdzitowiecki K, White GW (1997) Digenean, Monogenean and CestodeInection of inshore fish at the South Orkney Islands. Acta Parasitologica, 42(1), 18-22.

Zimmer C (1920) Die Cumaceen der Schwedischen Sudpolarexpedition, In: Schwedische Sudpolar-Expedition 1901-1903, 6, 1-16.
